# Gaining Insights into Aggressive Behaviour in Autism Spectrum Disorder Using Latent Profile Analysis

**DOI:** 10.1007/s10803-019-04129-3

**Published:** 2019-07-10

**Authors:** Matthew O. Sullivan, Louise Gallagher, Elizabeth A. Heron

**Affiliations:** Department of Psychiatry & Neuropsychiatric Genetics Research Group, School of Medicine, The Trinity Centre for Health Sciences, Trinity College Dublin, Dublin 8, Ireland

**Keywords:** Autism spectrum disorder, Aggression, Latent profile analysis, IQ, Anxiety, Child behaviour checklist

## Abstract

Aggressive behaviour is a significant issue for individuals with autism spectrum disorder (ASD), yet our understanding is limited compared to aggression in typically developing populations. This study examined behavioural, adaptive and cognitive data provided by the Simons Simplex Collection (N = 2184) to identify behavioural subgroups in children and adolescents with ASD using latent profile analysis. Results showed five subgroups that differed with regards to behavioural severity, IQ and adaptive behaviour. In two profiles with higher aggression, individuals had greater comorbid anxiety symptoms and attentional deficits and also differed in adaptive behaviour and IQ. These results identify potentially important avenues for research in aggressive behaviour in ASD.

Autism spectrum disorder (ASD) is characterised by highly variable cognitive ability and adaptive function, as well as diverse comorbid behavioural symptoms that can be impairing. Aggression is a particularly impactful and limiting co-occurring behaviour. Reported prevalences of aggression in ASD are 35–50% (Kanne and Mazurek 2011; Mazurek et al. 2013). It poses a significant challenge to caregivers and clinicians (Baker et al. 2002) and is strongly associated with psychiatric hospitalization (Mandell 2008) and adherence to psychotropic medication (Logan et al. 2014). Aggression also limits independence, community engagement and the capacity for fostering relationships (Benson and Aman 1999).

Strong predictors of aggression in typically developing (TD) individuals from longitudinal data include harsh parenting practices, low parent education, low IQ and male sex (NICHD Early Child Care Research Network 2004). Consistently high levels of aggression in childhood are predictive of future delinquent behaviour, difficulties in emotion regulation and impaired peer relationships (Loeber et al. 1998; Moffit et al. 1996). While it is known that aggression is associated with poor outcomes in ASD, there is, unfortunately, a deficit of longitudinal data in ASD cohorts from which robust predictors could be identified.

A number of large cross-sectional studies investigated group level predictors of aggression in ASD. Known predictors of aggression in TD populations (e.g. low IQ) were not predictive in a large ASD sample of children and adolescents (N = 1380), derived from the Simons Simplex Collection (SSC) (Kanne and Mazurek 2011). Autism associated symptoms, namely autism severity, intellectual functioning or adaptive behaviour, were also not associated. Separately, self-injurious behaviour, sleep problems and sensory issues were strong predictors of parent-reported physical aggression towards others in an analysis of data from the Autism Treatment Network (ATN; N = 1584) (Mazurek et al. 2013). While these studies offer some evidence for predictors of aggression, the role of IQ (Hill et al. 2014) and autism severity (Dominick et al. 2007) in mediating aggression remains unclear.

Comorbid anxiety was correlated with aggression in ASD in a few small studies (Gotham et al. 2013; Panju et al. 2015). It is unclear what factors mediate this relationship and if they interact. A three-way interaction between IQ, social understanding and aggression predicted anxiety in young children with ASD (Niditch et al. 2012). Paradoxically, low and high levels of social anxiety predicted aggression in individuals with high-functioning ASD (Pugliese et al. 2013). This may suggest that impaired behavioural inhibition mediates both low social anxiety and increases risk of aggression.

Cognitive inflexibility, or an inability to shift attentional focus, has also been identified as a trigger for aggression (Pugliese et al. 2014). It was associated with greater autism severity and also with an increased tendency to ruminate on angry cognitions resulting in aggressive outbursts. This contrasts with an absence of a relationship between aggression and autism severity previously reported by Kanne and Mazurek (2011). Given the inconsistencies, alternative approaches that take account of phenotypic variability, such as anxiety, IQ, adaptive functioning and autism symptoms, may identify other factors that contribute to comorbid aggression in ASD. This may enhance our understanding of the behavioural mechanisms underpinning aggressive outbursts.

Hypothesis free approaches can identify behavioural subgroups that are more likely to engage in aggressive behaviour. Latent profile analysis (LPA), which is also known as latent class cluster analysis (Vermunt and Magidson 2002) and finite mixture modelling (McLachlan and Peel 2000), is one such statistical approach that can uncover related cases from continuous data. LPA describes heterogeneity in a given sample using a finite number of discrete profiles. It identifies an unobserved latent variable, which signifies profile membership (Muthén and Muthén 2000). Individuals in these profiles (or subgroups) have relatively similar responses across the variables of interest, known as manifest variables.

LPA has been used previously to identify sensory subtypes in pre-school children with ASD (Tomchek et al. 2018) and differences in executive function between children with ASD and attention deficit/hyperactivity disorder and TD children (Dajani et al. 2016). It was also used to define profiles of personal and social coping among parents of children with ASD (Zaidman-Zait et al. 2018). To date, no study investigated behavioural subgroups in ASD including aggressive behaviour. Clinically relevant co-occurring issues may guide future studies on aggression in ASD and therefore investigation is warranted.

The aim of this study is to describe the heterogeneity of behavioural issues in the SSC linked to aggression in ASD using LPA. Manifest variables included in the LPA were selected based on previously reported relationships with aggression in ASD.

## Methods

The Simons Simplex Collection (SSC) is a national autism genetics repository based in North America that combines genotype data with extensive phenotype information (Fischbach and Lord 2010). Phenotype data include measures of cognitive and adaptive function, autism and common comorbid behavioural symptoms (www.sfari.org). The study design emphasised the inclusion of ‘singletons’, i.e. families with only one affected proband to increase the discovery rate of rare genetic mutations or copy number variants. Exclusion criteria were non-verbal mental age below 18 months, having a first degree relative with autism and having conditions known to cause autism (e.g. Fragile X Syndrome, low birth weight, extreme prematurity or severe neurological deficits). As a publicly available, extensively phenotyped dataset, it provides a useful resource to also investigate broader clinical questions.

The SSC dataset consisted of 2857 individuals with ASD (SSC Version 15 Phenotype Data). We included individuals who had clinical data from the School-Age form (ages 6–18) of the Child Behaviour Checklist (CBCL) that includes aggressive behaviour (see below). We excluded individuals who did not have this data. We excluded individuals who had data on the Pre-School form (ages 1.5–5) of the CBCL, as it differs at an item level in its assessment of aggression. Our derived sample included 2184 individuals, with a median age of 9.58 years (range = 5.67–18) and a mean IQ of 81.17 (SD = 27.96).

## Latent Profile Analysis Manifest Variables

The variables listed below were chosen because they were associated with aggression in previous studies. IQ, autism severity and adaptive behaviour have all been linked to aggression previously (Dominick et al. 2007; Farmer et al. 2015; Jang et al. 2011), though the reported associations are heterogeneous (Kanne and Mazurek 2011; Mazurek et al. 2013). Anxiety and attention deficits were also previously associated with aggression (Gotham et al. 2013; Lawson et al. 2015), as were self-injurious, sameness and ritualistic behaviour (Kanne and Mazurek 2011). The Irritability and Hyperactivity subscales of the Aberrant Behaviour Checklist were also previously highly correlated with the CBCL aggressive behaviour subscale (Kaat et al. 2014).

### Child Behaviour Checklist (CBCL)

The CBCL (Achenbach and Rescorla 2001) is a parent-report measure of emotional and behavioural functioning over the preceding 6 months. The CBCL Syndrome scales: aggressive behaviour, anxious/depressed and attention problems were included in our analysis. Each scale produces a T score, indicating normal to clinically relevant behaviours. Scores greater than 70 indicate the behaviour is of clinical relevance.

### Intelligence Quotient (IQ)

The SSC clinical data contains multiple IQ measures of cognitive ability and multiple measures are included for some individuals. The Differential Ability Scales, 2nd Edition (DAS-II; Elliott 2007) was the commonest measure (92%). Other measures included the Mullen Scales of Early Learning (Mullen 1995) (8%), the Wechsler Intelligence Scales for Children, 4th Edition (Wisc-IV; Wechsler 2003) (2%) and the Wechsler Abbreviated Scale of Intelligence (WASI; Wechsler 1999) (5%). SSC researchers computed a standardized IQ variable in the SSC to account for multiple IQ measures, of which 20% of our sample (n = 439) had non-standardised, ratio IQs because their scores were outside of standard ranges for deviation scores (see Kanne and Mazurek (2011) for a description of ratio IQs). We included both standardised and ratio IQs in the analysis.

### Autism Severity

The Autism Diagnostic Observation Schedule Calibrated Severity Score (ADOS CSS; Gotham et al. 2009; Lord et al. 2000) was included as a measure of autism severity. This is a standardized measure of autism severity that accounts for varying verbal ability and different chronological ages based on the use of different ADOS modules. We computed CSS values for individuals who completed module 4 based on the now published algorithm (Hus and Lord 2014). We excluded a small number of individuals who had CSS values below “4” (n = 5), which are scores indicative of “non-spectrum” and not fitting the inclusion criteria for the SSC.

### Vineland Adaptive Behaviour Scales 2nd Edition (VABS-II)

The VABS-II (Sparrow et al. 2005) is a semi-structured informant-based interview completed with parents/caregivers that measures an individual’s adaptive functioning. A number of domains are assessed, daily living skills, communication and socialization, and a standard score for each can be computed. We included the latter two in the analysis due to previous reported associations with aggression.

### Aberrant Behaviour Checklist (ABC)

The ABC (Aman et al. 1985) is a 58 item parent/caregiver completed questionnaire with subscales for irritability, lethargy, stereotypy, hyperactivity and inappropriate speech. We included irritability and hyperactivity in our analysis.

### Repetitive Behaviour Scale-Revised (RBS-R)

The RBS-R (Lam and Aman 2007) is a parent/caregiver completed questionnaire that assesses ASD associated repetitive behaviours. The subscales are ritualistic, sameness, self-injurious, ritualistic, stereotyped and compulsive behaviour. We included ritualistic, sameness and self-injurious behaviour in our analysis.

## Latent Profile Analysis

LPA is a person-centred statistical technique that facilitates the identification of groups of individuals that display similar response patterns, profiles, for a set of continuous variables (Berlin et al. 2014). LPA assigns probabilities to each individual for membership of the resulting groups, where each group has its own profile. Individuals have some probability for membership of the subgroups—this reflects the varying degree of certainty with regards to subgroup membership (Asparouhouv and Muthén 2007; Muthén 2001). The analysis estimates the proportion of individuals belonging to the different subgroups. The parameters of the LPA model are estimated using the individuals’ observable scores on each of the manifest variables and by fixing the number of profiles. Multiple LPA models, with different numbers of profiles, can be compared using measures of fit to determine the most appropriate LPA model for the data.

Latent profile analysis was implemented via the statistical program R (R Core Team 2018) using the tidyLPA package (Rosenberg et al. 2018). To identify the model with the optimal number of profiles, we examined the Bayesian Information Criterion (BIC; Schwarz 1978) values across models and performed a bootstrapped likelihood ratio test (BLRT; McLachlan and Peel 2000). The BIC examines a model’s parameters and provides guidance on which model captures the complexity of the data while still remaining as succinct as possible (i.e. fewer profiles). The BIC has superior performance in indicating model fit relative to other goodness of fit measures (Nylund et al. 2007; Yang 2006). The BLRT provides a measure of model fit and a p value for each profile that is added to the model. These criteria inform the choice of a final model to best describe the data. However it is possible to select a final model with greater/fewer profiles if the number of suggested models do not add to the conceptual or theoretical understanding of the group patterns (Vaillancourt et al. 2017). Separate to model fit indices, we also examined model entropy after a final model was chosen. Model entropy is an overall measure of the performance of a model in predicting profile membership (Muthén and Muthén 2000). Entropy values range from 0 to 1; higher values indicate superior predictive power (Celeux and Soromenho 1996).

## Results

A full range of data were available for 2079 individuals on the manifest variables (see Table 1), where the LPA algorithm in tidyLPA that was used only included those individuals who did not have missing data. We examined model fit statistics for two to five profiles (see Table 2). A 4-profile solution had the lowest BIC; however, we chose a 5-profile solution as this model provided the most clinically meaningful distribution of groups. All solutions returned a significant BLRT value, indicating models with greater number of profiles were the preferred solutions. However, the 5-profile solution identified an additional group with higher mean scores for the CBCL aggressive behaviour subscale. Despite a slightly higher BIC value, clinically, this model had more utility in furthering our understanding of aggression in our sample. The 5-profile model identified two distinct subgroups characterised by higher aggression scores, allowing us to explore aggressive behaviour in the context of other behavioural issues and reflect on potential relationships. Model entropy for the 5-profile solution was estimated to be 0.89, indicating that the model classified individual cases with high certainty (see Table 2). Mean scores for each manifest variable for each profile can be observed in Table 3.

**Table 1:** Demographic data for dataset with full range of data (N = 2079), including data pertaining to manifest variables

Variable	N	Median (range)	Mean (SD)
SSC individuals with ASD	2857	–	–
SSC individuals with clinical data on School-Age form (6–18) of CBCL including aggressive behaviour	2184	–	–
Complete cases on selected manifest variables	2079	–	–
Age	2079	115 (5.67–18)	123.3 (37.45)
Gender	M = 1798; F = 281	–	–
IQ	2079	85 (7–167)	81.52 (28.46)
VABS communication	2079	75 (30–132)	75.72 (13.67)
VABS socialisation	2079	70 (34–117)	69.98 (12.32)
ADOS CSS	2079	7 (4–10)	7.47 (1.69)
CBCL aggressive behaviour	2079	58 (50–97)	59.53 (9.1)
CBCL anxious/depressed	2079	57 (50–98)	59.49 (9.17)
CBCL attention problems	2079	66 (50–100)	66.79 (10.15)
ABC irritability	2079	10 (0–45)	11.35 (8.73)
ABC hyperactivity	2079	15 (0–47)	16.08 (10.34)
RBS-R ritualistic behaviour	2079	4 (0–18)	5.12 (3.98)
RBS-R self-injurious behaviour	2079	1 (0–21)	2.14 (2.86)
RBS-R sameness behaviour	2079	7 (0–32)	8.03 (6.09)

**Table 2:** Fit statistics and model entropy for 2- to 5- profile solutions

Profile solutions	Profile sizes	BIC	BLRT	Entropy
2	1, n = 801	159,842	1483.85*	0.94
	2, n = 1278			
3	1, n = 664	159,099	1207.08*	0.92
	2, n = 893			
	3, n = 522			
4	1, n = 282	158,499	927.79*	0.91
	2, n = 725			
	3, n = 641			
	4, n = 431			
5	1, n = 406	158,701	624.87*	0.89
	2, n = 243			
	3, n = 502			
	4, n = 520			
	5, n = 408			

*p value < 0.05

**Table 3:** Mean scores for each manifest variable by latent profile (N = 2079)

	Profile 1 (n = 406)		Profile 2 (n = 243)		Profile 3 (n = 502)		Profile 4 (n = 520)		Profile 5 (408)	
	Mean (SD)	Range	Mean (SD)	Range	Mean (SD)	Range	Mean (SD)	Range	Mean (SD)	Range
IQ	94.22 (21.55)	30–167	83.31 (27.09)	19–155	96.22 (18.18)	48–156	65.68 (29.88)	10–134	69.93 (28.38)	7–148
VABS communication	81.98 (12.61)	59–132	77.12 (13.6)	40–120	80.42 (10.36)	57–118	68.42 (12.48)	33–110	72.19 (14.4)	30–112
VABS socialisation	75.42 (10.37)	46–106	73.37 (13.48)	40–112	72.8 (9.87)	45–117	62.96 (11.64)	34–103	68.03 (12.43)	36–108
ADOS CSS	7.25 (1.77)	4–10	7.39 (1.68)	4–10	7.47 (1.71)	4–10	7.5 (1.64)	4–10	7.7 (1.62)	4–10
CBCL aggressive behaviour	54.97 (4.76)	50–68	50.11 (0.31)	50–51	64.64 (9.3)	50–97	65.2 (8.98)	50–94	56.19 (5.69)	50–78
CBCL anxious/depressed	60.99 (5.88)	50–80	51.6 (2.44)	50–60	66.23 (9.03)	50–98	62.66 (8.84)	50–94	50.39 (0.49)	50–51
CBCL attention problems	62.65 (7.14)	50–90	58.69 (6.5)	50–83	68.75 (8.76)	50–96	73.22 (11.17)	50–100	65.11 (8.92)	50–100
ABC irritability	5.86 (3.84)	0–17	2.06 (1.97)	0–8	14.96 (7.47)	0–36	18.34 (8.89)	0–45	9 (6.09)	0–27
ABC hyper-activity	9 (5.19)	0–25	5.58 (4.48)	0–19	19.86 (9.38)	0–45	22.68 (9.94)	1–47	16.29 (9.13)	0–42
RBS-R ritualistic behaviour	3.6 (2.54)	0–13	2.42 (2.23)	0–10	6.12 (3.98)	0–18	7.54 (4.5)	0–18	3.93 (3.08)	0–13
RBS-R self-injurious behaviour	1.41 (1.58)	0–7	0.19 (0.39)	0–1	0.92 (1.06)	0–4	5.23 (3.71)	0–21	1.59 (1.78)	0–8
RBS-R sameness behaviour	4.78 (2.9)	0–13	2.61 (2.28)	0–11	10.18 (5.58)	0–28	12.51 (6.76)	0–32	6.16 (4.31)	0–21

**Fig. 1:**
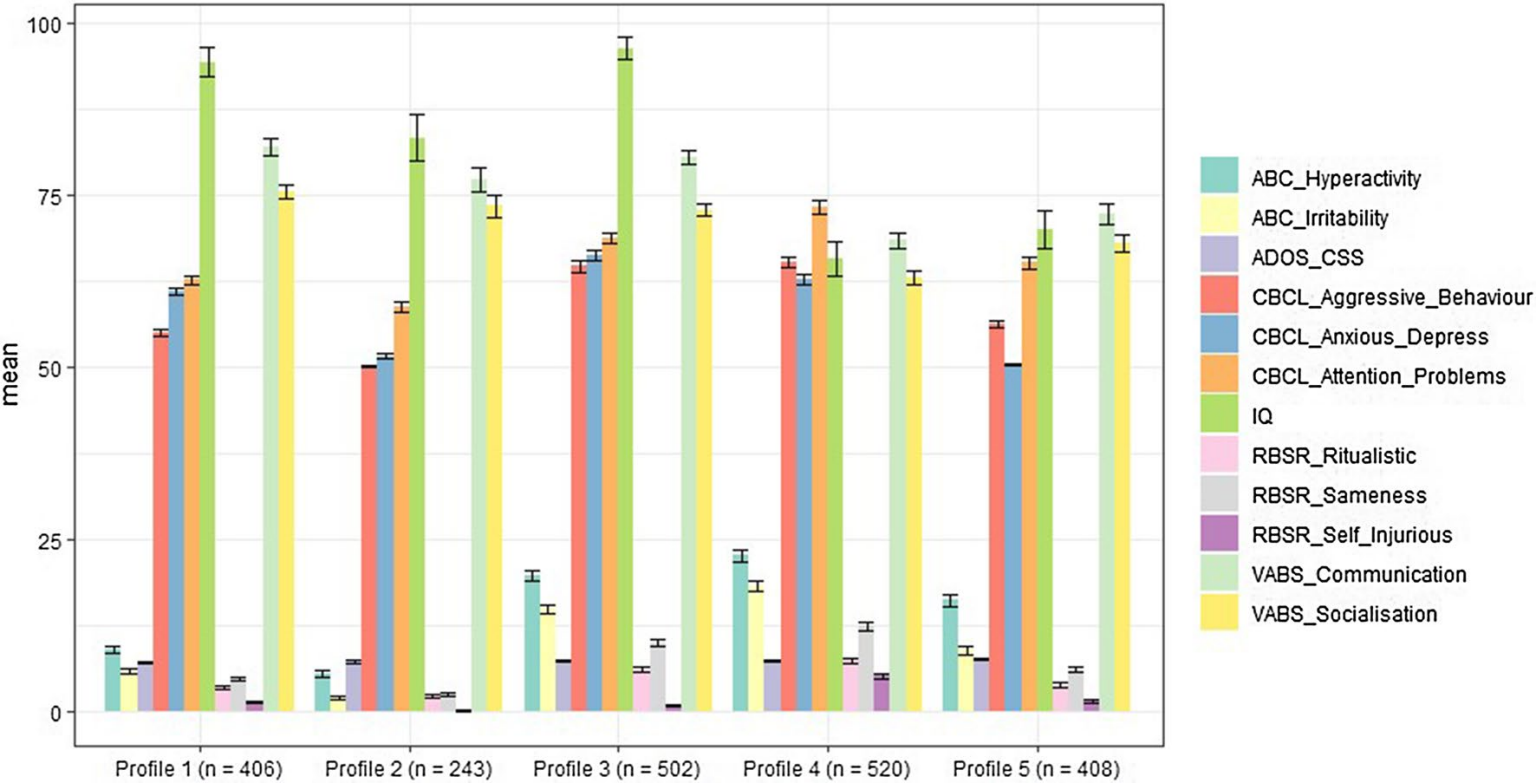
Manifest variables’ mean scores with standard errors across the five profiles

The 5-profile solution described five behavioural subgroups in the SSC (see Fig. 1). We characterised the subgroups by behavioural severity, denoting subgroups with “low”, “mid” and “high” labels with regards to the CBCL, ABC and RBS-R subscale scores. Terms such as “higher” and “lower” were used as an indicator of relative behavioural severity between groups. These labels differentiate individuals in our sample relative to one another and do not indicate different levels of clinical significance. The VABS domains, IQ and ADOS CSS also provided more information in distinguishing groups in terms of cognitive/adaptive functioning and severity of ASD symptoms.

We identified a “Low Severity” group (Profile 1); another “Low Severity” group (Profile 2); two “High Severity” groups (Profiles 3 and 4); and a Mid Severity” group (Profile 5). The mean posterior probabilities across the five profiles were high, ranging from 0.85 to 0.94, indicating individuals were classified into their respective profiles accurately.

Profile 1 (Low Severity; n = 406) was characterised by individuals with higher mean IQ and a lower severity behavioural profile. Mean scores for CBCL anxious/depressed and attention problems were lower than all other profiles with the exception of Profile 2 which showed the lowest behavioural severity of all five profiles.

Individuals in Profile 2 (Low Severity; n = 243) generally showed the lowest severity in behavioural presentations with lowest mean scores across the CBCL, ABC and RBS-R subscales. These individuals had mean IQ scores that were higher than Profiles 4 and 5 but lower than the two higher IQ profiles (Profiles 1 and 3).

Profile 3 (High Severity; n = 502) was characterised by individuals with a more complex behavioural profile. Mean scores across the ABC, CBCL and RBS-R subscales were higher than Profiles 1, 2, 4 and 5.

We observed the most severe behavioural profile in individuals in Profile 4 (High Severity; n = 520). Mean scores for the ABC, CBCL and RBS-R subscales were higher in this profile relative to the less severe profiles. Individuals in this profile presented with higher scores on RBS-R subscales and CBCL attention problems compared to individuals in Profile 3.

Individuals in Profile 5 (Mid Severity; n = 408) generally presented with lower mean scores for the behaviour measures, CBCL, ABC and RBS-R subscales, relative to other more severe profiles. However, these individuals presented with higher ABC hyperactivity and CBCL attention problems mean scores relative to other profiles, accounting for the label “Mid Severity”.

Autism severity, as measured by the ADOS CSS, did not clearly differ between the profiles. Three profiles with higher IQ (Profiles 1–3) also had higher mean scores for the VABS Communication and Socialisation domain. The reverse was observed for profiles where IQ was lower (Profiles 4 and 5).

**Fig. 2:**
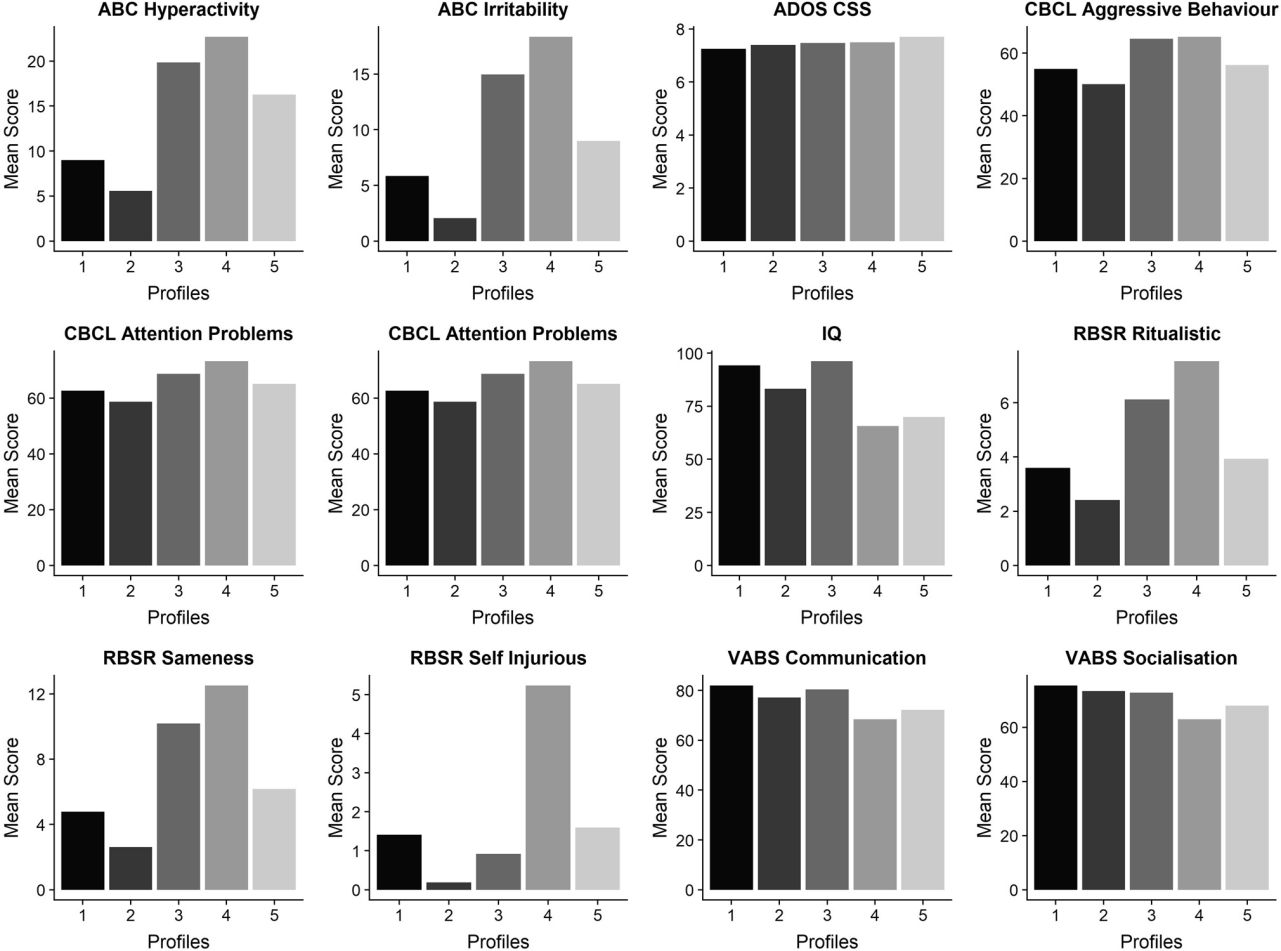
Manifest variables’ mean scores across latent profiles, plotted by variable

Profiles 3 and 4 had elevated mean scores for aggression, anxious/depressed symptoms, attention problems and RBS-R sameness and ritualistic behaviour. We found, however, that CBCL attention problems; ABC irritability and hyperactivity; and RBS-R self-injurious behaviour were more pronounced in Profile 4 compared to Profile 3. Profile 4 also represented individuals with lower levels of cognitive ability (Fig. 2).

## Discussion

Aggression is a significant co-occurring symptom in ASD that is significantly impairing and impactful. Our aim was to provide a deeper understanding of the additional clinical characteristics that may accompany aggression to inform further research and provide useful clinical insights. To this end we used latent profile analysis, which is a hypothesis free approach that identifies profiles with similar response patterns across the variables of interest. Based on our analysis, five profiles were identified with varying levels of aggression severity and additional behavioural characteristics. Our decision for a 5-profile solution to describe the data from the SSC was informed by examination of fit statistics and by the clinical applicability of the model. We identified two subgroups with profiles characterised by higher mean scores for CBCL aggressive behaviour. We also observed greater inattention, hyperactivity, anxiety and behavioural rigidity in these two subgroups.

We observed raised mean scores for anxiety and aggression in Profiles 3 and 4. Other studies examining anxiety in ASD have reported social anxiety predicts aggressive behaviour (Pugliese et al. 2013), three-way interactions between IQ, aggression and social understanding predict anxiety (Niditch et al. 2012) and that higher anxiety has been associated with increased aggression (Gotham et al. 2013; Panju et al. 2015). It has been reported that executive function deficits (e.g. issues with inhibition, working memory, planning and flexibility) are associated with anxiety and aggression in ASD and may serve as a pathway to comorbid psychopathology (Lawson et al. 2015; Visser et al. 2014). Potentially, the comorbidity of these symptoms observed in the two subgroups (Profiles 3 and 4), who also showed increased attentional issues and hyperactivity, may be characteristic of executive function impairments influencing aggressive presentations. Notably, Lawson et al. (2015) note differences between pathways to aggression in children with ASD and children with ADHD, stating that ASD children may be aggressive due to issues in flexibility whereas issues with behavioural inhibition may drive aggression in ADHD. We observed higher RBS-R sameness behaviour in these subgroups with higher aggression, which may be representative of Lawson et al.’s (2015) findings. The absence of any measure of executive functioning for probands in the SSC precluded any investigation of these impairments, however future studies may examine this further.

Individuals in Profile 3 had higher mean IQ and Communication and Socialization scores on the VABS relative to those in Profile 4 in this analysis. A relationship of IQ and adaptive behaviour with aggression was not reported in previous studies (Hill et al. 2014; Kanne and Mazurek 2011) and our results indicate that individuals with low or high scores in IQ and VABS Communication and Socialisation also had aggressive behaviour. Farmer et al.’s (2015) study reported an association between lower IQ/adaptive behaviour and increased physical aggression compared with increased verbal aggression in those with higher IQ/adaptive behaviour. While individuals in Profile 3 and 4 have higher aggression relative to the remainder of the sample, they may differ in their type of aggression displayed. Two predominant subtypes of aggression are proposed more generally, labelled *proactive* and *reactive* aggression (Farmer et al. 2016; Pouw et al. 2013). These subtypes are based on deconstruction of aggression through more in-depth phenotyping of aggressive behaviour. We were not able to explore this to the same extent in this analysis, as the publicly available SSC data do not release item-level CBCL data that would be required for more detailed characterisation of aggression subtypes. Additionally, individuals with intellectual disability were under-represented in this sample. Were there a greater balance of individuals with low and high IQ, individuals may have been distributed differently across the subgroups. However, further investigation of aggression subtypes in ASD will require careful phenotypic characterisation that ultimately may be important for intervention.

Though our analysis yielded distinct and seemingly meaningful behavioural subgroups, it is important to acknowledge the limitations of our analysis. Chiefly, these data were cross sectional in nature. Therefore, we were unable to evaluate the potential effects of age and development on the subgroups. Currently, there is a lack of longitudinal research into aggression in ASD, which might provide clearer insights into the development of aggressive behaviour in ASD over time. Aggression can be age-appropriate in all young preschool children (Connor 2002), however it is still unclear what constitutes *normative* levels of aggression in ASD. Longitudinal studies of aggression would provide more understanding of characteristics and the stability of the behavioural trajectories of these subgroups over time.

Additionally, the lack of validity data for the identified subgroups presents a challenge in determining the significance of these results. This study moved beyond ASD symptomology to group children and adolescents, however, without any replication datasets or external validation criteria we were unable to validate the behavioural subgroups. Future studies may address this by attempting to replicate the results in an independent sample and externally validating the latent classes via variables not included in the original analysis.

The definition of aggression used here was the CBCL Aggressive Behaviour Problems scale. This scale includes items relevant, but not specific to, *physical* aggression, therefore we cannot conclude whether the observed relationships pertain to specific subtypes of aggression. Definitions of aggression are inconsistent across studies (Farmer and Aman 2011) and, as a consequence, the existing literature is difficult to integrate and interpret. The development of a measure sensitive to aggression subtypes—the *Children’s Scale for Hostility and Aggression: Reactive/Proactive* (Farmer and Aman 2010; Farmer et al. 2016)—will be useful to provide more standardised definitions and characterisation of both proactive and reactive aggression. Additionally, analyses distinguishing the topography of aggressive behaviour (i.e. physical and verbal aggression) will provide a more nuanced and insightful characterisation of aggression. Improvements to our understanding of different forms of aggression are necessary to determine the underlying mechanisms.

We acknowledge there may be broader contextual influences on aggression in ASD, such as classroom and family variables. Aggressive behaviour has been linked to classroom removal (Tsakanikos et al. 2007) and is a major stress for parents (Hodgetts et al. 2013)—a child’s environment may impact on how and when they express aggressive behaviour. These contextual factors are important to consider when evaluating aggressive behaviour, however such factors were beyond the scope of the data available to us from the SSC.

Additionally, there may be a limit to how generalizable our results are to the broader ASD population. The SSC was designed to collect information from families with only one child with autism. It is plausible that this could influence the types of behavioural presentations observed in this cohort. Howe et al. (2014) note that differences in ascertainment protocols between large-scale datasets (e.g. Autism Treatment Network, Autism Genetics Resource Exchange and the SSC) impacted reported gender differences across the datasets’ measures. Fischbach and Lord (2010) note the original SSC dataset had little intellectual disability and, in the current dataset we derived our sample from, < 30% of individuals had an intellectual disability. This may reflect ascertainment biases in the SSC and the consequently small number of individuals displaying clinical CBCL aggressive behaviour scores (14%). As a community sample, the profiles observed here may show fewer behavioural comorbidities than a clinically ascertained sample.

Despite these limitations, these subgroups appear clinically meaningful. The subgroups emphasise the importance of further examining aggression in the context of other behavioural issues. This is clinically relevant and may in time inform more person-centred interventions for this common behavioural comorbidity. The identification of clinical predictors early in life could support preventative strategies for the development of aggression. Our focus on broader behavioural characteristics is relatively unique and may help to explain behavioural mechanisms, and potentially neurobiological differences, that drive aggressive behaviour in ASD. This would be most powerful in the context of longitudinal data, which would also facilitate the identification of predictors of aggression over time. However, the present study offers an alternative approach to examining aggression and indicates potentially relevant constructs of interest for future studies.
